# Advances in the eradication of foot-and-mouth disease in South America: 2011–2020

**DOI:** 10.3389/fvets.2022.1024071

**Published:** 2023-01-09

**Authors:** Alejandro Mauricio Rivera, Manuel Jose Sanchez-Vazquez, Edviges Maristela Pituco, Lia Puppim Buzanovsky, Monica Martini, Ottorino Cosivi

**Affiliations:** Pan American Center for Foot-and-Mouth Disease and Veterinary Public Health (PANAFTOSA/VPH), Pan American Health Organization, Regional Office for the Americas of the World Health Organization, Rio de Janeiro, Brazil

**Keywords:** foot-and-mouth disease, disease elimination, hemispheric program for the eradication of foot-and-mouth disease (PHEFA), foot-and-mouth vaccination, foot-and-mouth control program

## Abstract

For more than 70 years, the countries of South America have been attempting to eliminate foot-and-mouth disease (FMD), but a regional strategy had not been established by all the affected countries until 1988. The Action Plan 1988–2009 of the Hemispheric Program for the Eradication of Foot-and-Mouth Disease (PHEFA 1988–2009) resulted in an FMD-free status in 88.4% of the bovine population of South America. However, countries of the Andean sub-region maintained an FMD endemic. In addition, sporadic outbreaks in vaccinated cattle populations have been reported in countries of the Southern Cone, endangering the disease-free status in these countries. Within this context, the PHEFA 2011–2020 was approved to eliminate FMD from the subcontinent, and this review describes the most important milestones during its execution. FMD in Ecuador and sporadic outbreaks in the Southern Cone sub-region were effectively eliminated. The outbreaks that occurred in Colombia in 2017 and 2018 were successfully controlled. The type C virus was removed from the vaccines in use in most countries, based on a risk assessment. This review also describes the progress made by the countries advancing toward official recognition as FMD-free in all their territories, with Bolivia, Brazil, and Peru leading the progressive suspension of vaccination to achieve FMD-free status without vaccination. Consequently, at the end of PHEFA 2011–2020, Venezuela was, and still is, the only country in the region whose control program has suffered setbacks, and no evidence has suggested that the transmission and infection of the bovine population have been eliminated. At the end of 2020, a new PHEFA Action Plan 2021–2025 was approved with a five-year horizon, to complete the eradication of the disease in the Americas.

## Introduction

Foot-and-mouth disease (FMD) was introduced in South and North America in 1879, with two different outcomes. While in North America the sporadic occurrence of outbreaks resulting from imports of animals and products was confronted with an elimination strategy by stamping-out and quarantining, in South America the infection spread to bovine populations in all affected countries (i.e., Argentina, Bolivia, Colombia, Chile, Brazil, Paraguay, Peru, Uruguay, and Venezuela) (1). This spread was enabled by the expansion of extensive bovine livestock farming, colonizing large territories in South America, leading to a very active livestock movement network (1).

**Figure 1:**
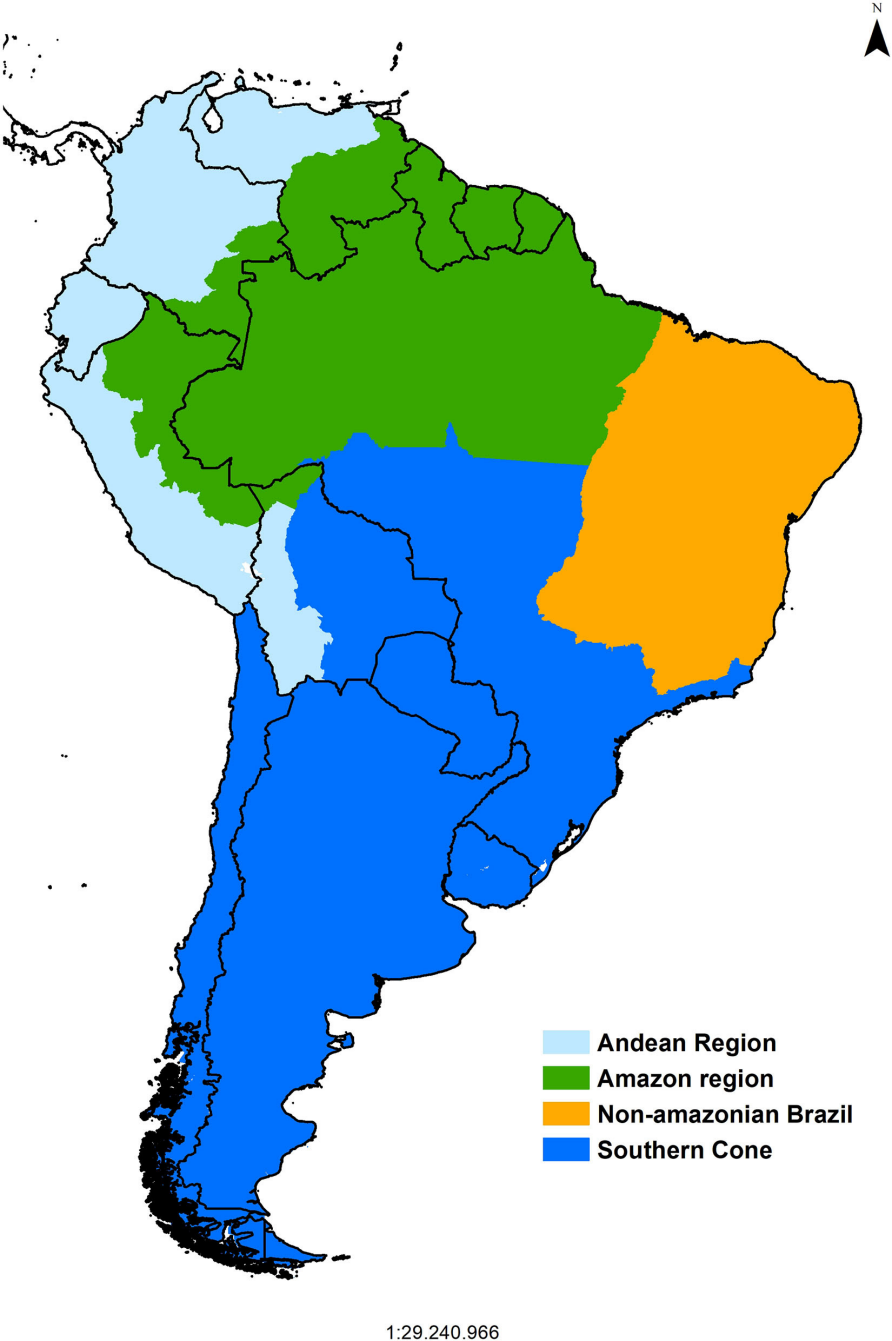
PHEFA South American sub-regions.

The Pan American Center for Foot-and-Mouth Disease, PANAFTOSA-PAHO/WHO established in 1951, which initially focused on laboratory diagnosis, characterization of FMD epidemiological areas, identification of the virus strains, development of vaccines, and delivering training and technical cooperation to the affected countries was a game changer for the control of FMD in South America. In the 1970s, the South American Commission for the Fight against Foot-and-Mouth Disease (COSALFA) was established as the high-level technical and political mechanism to coordinate national plans for the control of FMD in South American countries (1). The COSALFA delegates from each country (both from public and private sectors) meet annually to assess the progress made and address regional issues, such as establishing priorities for PANAFTOSA-PAHO/WHO technical cooperation (e.g., to focus on specific countries), technical recommendations (e.g., those to progress toward FMD status without vaccination or risk assessment), and logistics tools (e.g., vaccine banks). By the end of the 1980s, the Hemispheric Committee for the Eradication of Foot-and-Mouth Disease (COHEFA) was created, and it approved the Hemispheric Program for the Eradication of Foot-and-Mouth Disease (PHEFA), which provides a strategic framework to coordinate the eradication efforts of national plans in the six sub-regions of the American continent (1). The program relied on the extensive knowledge gained on the natural history of the disease and its determinants in South America, being characterized in four sub-regions (Figure 1). Bovine production systems and movement patterns determined the historically observed disease presentation and its dissemination; therefore, PHEFA promoted a control strategy based on reducing the susceptibility of bovine populations to the infection by means of systematic mass vaccination campaigns, together with strict animal movement control and response to outbreaks in all the affected countries of South America (1–3).

The first PHEFA Action Plan extended from 1988 to 2009, and although it did not eradicate the disease, it made significant progress: approximately 85% of the South American bovine population was recognized as free from FMD, with or without vaccination. Nevertheless, some areas still experienced FMD endemics (1). For example, in the Andean sub-region, regular FMD outbreaks were observed in Ecuador and Venezuela. Furthermore, the FMD-free status of Argentina, Bolivia, Brazil, and Paraguay, was suspended due to the sporadic occurrence of FMD outbreaks from 2002–2006, after the 2000–2001 epidemic (1), which jeopardized their FMD-free status with vaccination. Although the states of the northern region of Brazil, as well as the Amazon and Bolivian valleys exhibited a long period without FMD outbreaks, they had not demonstrated the absence of virus circulation, failing to achieve FMD-free status (1).

Therefore, in 2010, COHEFA approved a new action plan for 2011–2020 to complete the eradication process in South America, as the contribution of the Americas to the Global Foot-and-Mouth Disease Control Strategy, fostered by the Food and Agriculture Organization of the United Nations (FAO)/World Organization for Animal Health (WOAH) launched in 2012 (1, 4, 5). The involved countries seek international recognition of the progress made toward eradication within their territories through the WOAH free status recognition process (6). Recognition of FMD-free status by the WOAH reflects advances made by the countries in their wider capacity to control diseases of livestock and provides a tool to enable access to international markets.

**Figure 2:**
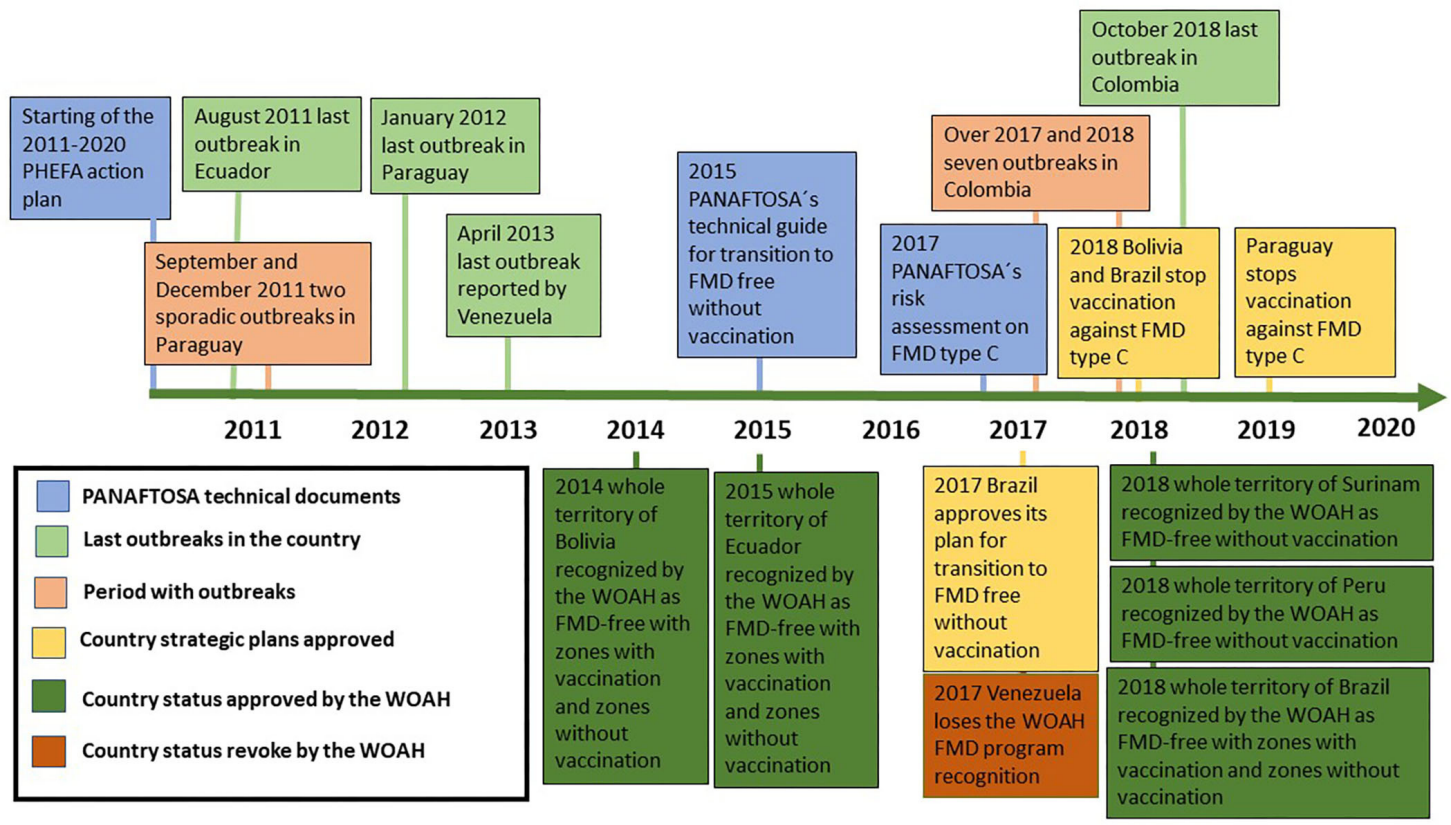
Timeline of key events and milestones during the implementation of PHEFA 2011–2020.

This review aims to describe the key milestones that characterized the implementation of the second PHEFA action plan between 2011 and 2020, namely, FMD elimination in Ecuador, FMD elimination in Paraguay following the 2011 outbreaks, FMD elimination in Colombia after the 2017 and 2018 outbreaks, the epidemiological situation in Venezuela, the evidence to not having experienced a type C outbreak since 2004 allowing the suspension of vaccination against this serotype, progress toward WOAH recognition of FMD-free status in more zones within the continent, and transitioning toward more zones achieving FMD-free without vaccination status. Figure 2 presents a timeline highlighting the key milestones that are addressed in this review.

## Elimination of FMD in Ecuador

By the end of the PHEFA Action Plan 1988–2009, Ecuador was experiencing epidemic outbreaks of FMD throughout the country, caused by type O virus, due to the poor implementation of vaccination campaigns and animal movement control (7).

In their phylogenetic analysis of the type O FMD viruses circulating in the Andean sub-region of South America in 2002 and 2008, Malirat et al. (8) characterized 11 different lineages. Virus isolates grouped within lineage 1 were mostly native to Ecuador, and some virus isolates collected from outbreaks that occurred in Colombia and Peru corresponded to virus incursions from Ecuador (8). Within lineage 1, nine subgroups were identified, corresponding to virus isolates collected during outbreaks that occurred in different provinces and years, with divergence values of 6–14% (8). Within this period, no virus isolates belonging to other lineages from other serotype O viruses circulating in the Andean sub-region were documented, suggesting that the occurrence of FMD in Ecuador resulted from endemic virus transmission in its bovine population along with epidemic cycles (8).

**Figure 3:**
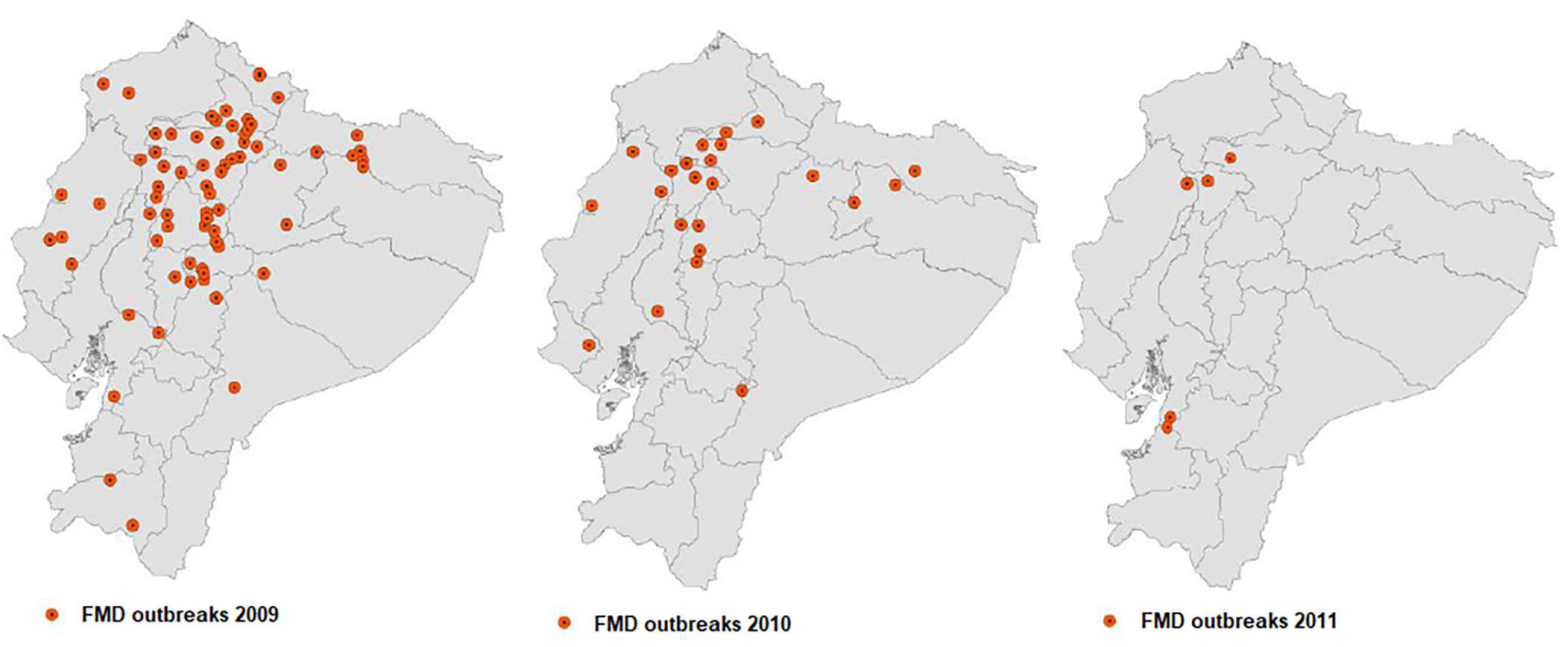
Location of the FMD outbreaks in Ecuador: 109 reported in 2009, 42 in 2010, and 5 in 2011.

The FMD epidemic observed from 2009 to 2010 prompted the response of international cooperation, led by PANAFTOSA-PAHO/WHO, which collaborated with the veterinary authorities of Ecuador in a full review of the FMD control program, which was under the responsibility of a private sector entity. The technical cooperation of PANAFTOSA led to a major change in the management, responsibilities, and control and monitoring mechanisms of the FMD program, complemented with good vaccination practices and farm registry management, which resulted in a rapid decrease in the incidence of FMD. The last five FMD outbreaks in Ecuador were documented in 2011 (7) (Figure 3).

A controversy emerged with *in vivo* and *in vitro* laboratory analyses performed on virus isolates obtained during the 2009 epidemic to predict the effectiveness of commercial vaccines against field virus strains. Maradei et al. (9) reported that *in vitro* vaccine matching studies, carried out by virus neutralization tests (VNTs), suggested a loss of protective response, which was supported by *in vivo* studies using the Protection against Podal Generalization test in cattle.

Duque et al. (7) observed that on the virus isolates from Ecuador in 2010, while the “r” values of the antigenic correlation between the field isolate and the strain of the vaccine were in the low range of the predictive scale for protection, the results of both the Expected Percentage of Protection (EPP) test developed by PANAFTOSA and the Protection against Podal Generalization test showed that protection was satisfactory (90%) in revaccinated animals but not in cattle that received just the first dose (approximately 50% of protection). PANAFTOSA (10) observed that the different methodologies and interpretations of vaccine matching studies explained the discrepancies in laboratory conclusions, but they were not consistent with the epidemiological situation observed in the field, both in the country and in the borders of neighboring countries. Therefore, decisions about changes in the composition of vaccines could not yet be made. As a result, biannual vaccination campaigns were supplemented for all cattle in continental Ecuador with booster vaccination for young animals on at-risk premises located in areas of extensive livestock farming, along with vaccine quality assurance and the implementation of good vaccination practices and effective control of animal movement (11).

In August 2011, the last case of FMD was documented, and the continental region of Ecuador achieved in 2015 disease-free status with vaccination, according to the WOAH, upon completion of serological studies showing the absence of virus transmission, while the Galapagos Islands achieved recognition as FMD-free without vaccination. Since then, Ecuador has been implementing serological surveys annually to check the immune status of vaccinated populations and confirm the absence of virus circulation to maintain its FMD-free status (12).

## The 2011 FMD outbreaks in Paraguay

At the start of PHEFA 2011–2020, the countries of the Southern Cone sub-region (i.e., Argentina, Bolivia, Chile, Paraguay, and Uruguay) had systematic vaccination programs in place and were recognized as FMD-free (1). However, in the previous period (1), sporadic recurrences of FMD outbreaks caused by the type O virus were observed in border territories of Southern Cone countries already recognized as free from FMD with vaccination, which affected Paraguay (2002 and 2003), Argentina (2003 and 2006), and Brazil (2005) (1).

Malirat et al. (13) described that the type O viruses responsible for these sporadic outbreaks from 2005–2006 in border areas of the Southern Cone countries not only corresponded to the Europe-South America topotype of the FMD virus but also showed a close phylogenetic (>90%) similarity. Therefore, these viruses were grouped within a single lineage with FMD type O viruses isolated during the epidemic outbreaks of 2000 that occurred in Brazil, Argentina, and Uruguay as well as in outbreaks that occurred in Brazil in 1998 and Bolivia in 2000, 2001, and 2003. This lineage was substantially different from that of the other circulating viruses in the Andean sub-region and in other geographic regions of South America.

An FMD outbreak was detected in September 2011 in a bovine herd in the department of San Pedro in central Paraguay. The virus was classified as type O and belonged to the Europe-South America topotype of the FMD virus, and phylogenetic analysis confirmed that it was the same lineage of previous isolations made in the Southern Cone sub-region (14, 15). The outbreak was controlled with measures including a stamping-out policy in addition to emergency vaccination in the control areas without the identification of secondary outbreaks associated with the index case. However, by the end of December 2011, a new infected herd was detected in the periphery of the controlled areas, which led to the reimplementation of sanitary measures for its control and elimination (16, 17). In both outbreaks, evident clinical signs of the disease were observed in young animals. The primary focus of those cases could not be determined, and the investigation showed no relationship between the two outbreaks. Nevertheless, the evidence that the virus acting in these outbreaks belonged to the same lineage of virus O circulating in the Southern Cone, at least since 1998, that had also been isolated in the outbreaks of 2002 and 2003 that had occurred in Paraguay, suggested that viral transmission was maintained in the vaccinated population due to the existence of endemic niches in the territory that were not detected by the surveillance system. Caporale et al. (18) suggested that when the proportion of immunized animals in a population does not reach a minimum value to block virus transmission, the herd immunity level is too high to enable an epidemic occurrence but too low to eliminate virus circulation, resulting in an endemic niche of infection, which is probably clustered in a particular production system or localized in marginal areas. This endemic niche could sporadically result in an FMD outbreak due to changes in the equilibrium between the virus and the population or when animals of these endemic niches are moved to areas where animals are not vaccinated or have low levels of vaccination coverage.

Following the end of the outbreak in March 2012, a full review of the national FMD program in Paraguay was carried out particularly of the vaccination program, with the support of PANAFTOSA-PAHO/WHO and the member countries of the Permanent Veterinary Committee of the Southern Cone (17). Several deficiencies and gaps detected in the national FMD program were addressed, which included booster vaccination targeted at all bovines under 1 year of age. Thus, as of 2012, the annual vaccination schedule included two general vaccination cycles targeted at all bovines and buffaloes, and one booster cycle for all young animals administered 30 days after the first general cycle. Maradei et al. (15) conducted tests that estimated the protection given by the vaccine strain, O_1_ Campos, from the vaccines in use against the virus isolated in Paraguay in 2011 and observed low protection (48.9%) 30 days post-vaccination in estimates from the EPP tests. However, the Podal Generalization tests performed 79 days after vaccination and 79 days after revaccination, showed an estimated protection of 75.0 and 87.5%, respectively. Furthermore, the Reference Laboratory of PANAFTOSA-PAHO/WHO estimated the immune coverage of the O_1_ Campos vaccine strain against the strain isolated in Paraguay in 2011 in the EPP test using ELISA-CFL to be 78.99% 30 days after vaccination and 99.70% 30 days after revaccination (14).

Annual FMD surveillance aimed at detecting the disease was also supplemented in 2014 with serological studies to determine the prevalence of post-vaccination antibodies and intended to estimate immune protection in different categories of bovines (19). Since then, these studies have been conducted annually throughout Paraguay to maintain a high immune level in the bovine population. As a result, these studies have improved vaccine coverage, encouraged good vaccination practices, and provided detailed information of the immune status of the population. As of 2012, no new cases of FMD had been detected in Paraguay or in the Southern Cone countries, demonstrating the elimination of infection and endemic niches that caused the sporadic outbreaks observed until 2011 (12).

## Outbreak of FMD in Colombia

Since Colombia achieved FMD-free status, it had experienced sporadic transboundary incursions of the FMD virus on the border with Venezuela in 2004 and 2008 and on the border with Ecuador in 2009. In 2011, a small zone in the northwest region of Colombia was recognized as FMD-free without vaccination, while the rest of the country was FMD-free with vaccination, except for a protection zone including parts of the departments bordering Venezuela. Since Ecuador achieved FMD-free status in 2015, the transboundary risk of FMD introduction was limited to the eastern border shared with Venezuela. Moreover, Colombia and Venezuela share a similar bovine husbandry system on both sides of the border.

**Figure 4:**
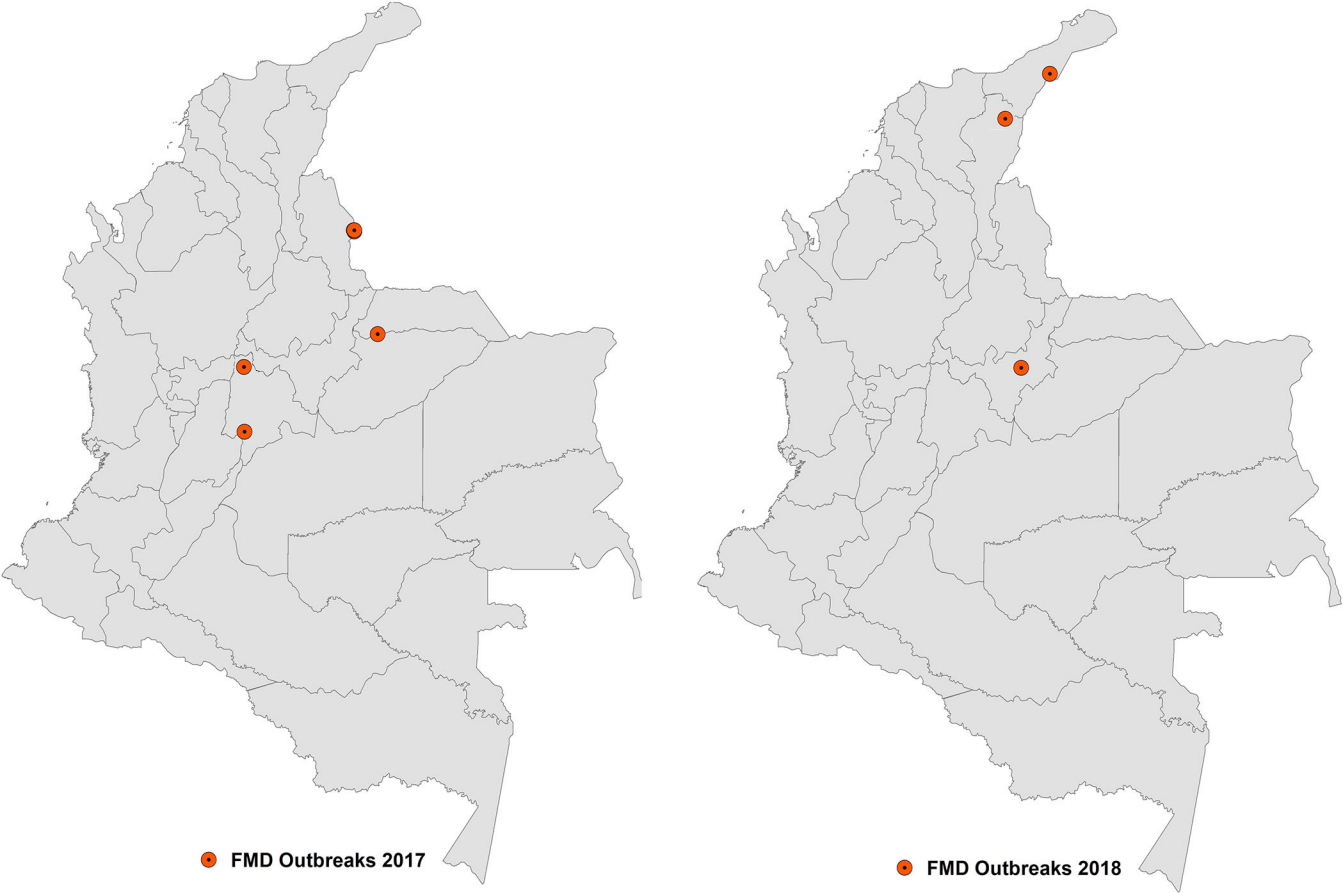
Location of the FMD outbreaks in Colombia in 2017 and 2018.

In June 2017, Colombia reported an FMD outbreak in the department of Arauca, bordering Venezuela. The investigation of this outbreak presumed that the source of infection was the smuggling of infected animals from Venezuela. In the same month, a second outbreak was reported in a mountain area in the department of Cundinamarca, in the center of the country, which affected several small herds. Later, in July, a third outbreak was confirmed in a small bovine herd in the same department, 134 kilometers from the second outbreak (20). The two outbreaks reported in the department of Cundinamarca were suggested to be related to contaminated meat products introduced by Venezuelan immigrants, which might have been used as swill feeding in pigs (20), although no clear evidence supported this hypothesis. The occurrence of FMD that year ended up with the detection of a fourth outbreak in the protection zone, very close to the border with Venezuela, which was also related to the illegal entry of animals from this country. Figure 4 illustrates the distribution of the 2017 and 2018 outbreaks that occurred in Colombia.

The outbreaks that occurred in Arauca and the protection zone were considered to be unrelated to each other or to those that occurred in Cundinamarca; instead, they were considered different virus incursions coming from the same country.

The phylogenetic analysis of virus isolates obtained from the four outbreaks conducted in the WOAH Reference Laboratory of PANAFTOSA-PAHO/WHO confirmed that all of these isolates belonged to lineage 6 of the type O virus, according to the classification of virus genotypes circulating in South America proposed by Malirat et al. (8). Specifically, lineage 6 isolates were identified in FMD outbreaks that occurred both in Venezuela and in bordering departments of Colombia between 2003 and 2009; therefore, the virus isolates of 2017 are consistent with a genotype that has been circulating in the north of the Andean sub-region, at least since 2003.

The outbreaks were controlled according to the standard measures applied by FMD-free countries with vaccination in South America: imposing quarantines and sanitary control zones, applying stamping-out to all animals in the affected herds followed by cleaning and disinfection, increasing surveillance for the detection of new cases, and performing an epidemiological investigation to determine the origin and the relationship between outbreaks. These measures enabled the establishment of a containment zone that included the three outbreaks in the FMD-free zone, which was recognized by WOAH in December 2017, enabling the official FMD-free status to be restored for the rest of the country (20).

In September 2018, a new outbreak was confirmed in the department of Boyacá in the containment zone caused by the type O virus. This recurrence caused the FMD-free with vaccination status of the country to be suspended. This outbreak could not be associated with a new virus incursion from Venezuela, and it was likely the result of remaining infection transmission within the containment zone. Additionally, in October of the same year, two new outbreaks were detected very close to the border with Venezuela in the departments of El César and La Guajira, both located outside of the containment zone. These two outbreaks were considered to be the result of two incursions of the FMD virus associated with the illegal entry of infected animals from Venezuela (20). Virus isolates obtained in the outbreaks of 2018 belonged to lineage 6, according to the classification proposed by Malirat et al. (8) and showed a high level of homology with isolates from 2017 (21).

The recurrence of the disease led to a full review of the FMD control and prevention strategy in Colombia. The information obtained through population immunity studies was key to the review. Post-vaccination monitoring of the whole bovine population located in the containment zone was carried out to assess its immune status. Although the serological study revealed an overall prevalence of animals protected against FMD type O virus of 78% (95% CI 77.0–80.0%), 5 to 6 months after the last vaccination cycle, small herds of some departments included in the containment zone showed a level of protection significantly lower than expected, which may have led to the establishment of niches facilitating virus transmission after reintroduction. Vaccine matching tests against isolates obtained in 2018 with EPP using the VNT and ELISA-CFL tests performed by the WOAH Reference Laboratory of PANAFTOSA-PAHO/WHO showed that the vaccine provided protection equivalent to 99.90% in a panel of sera obtained 30 days after vaccination and 99.99% in a panel of sera obtained 30 days after revaccination (21). After the second vaccination cycle of bovines conducted in October and November 2018, an additional vaccination cycle was conducted in January and February 2019 for the entire bovine population located in the departments where the FMD outbreaks occurred in 2017 and 2018.

In March and April 2019, four cross-sectional serological surveys were conducted on the bovine population. Three of them covered the entire vaccinated bovine population in the national territory with its official FMD-free status suspended, and one covered the protection zone. Tests to detect virus transmission and assess the apparent prevalence of FMD protective antibodies were performed. The selection of sampling units was carried out in two phases, and samples were randomly distributed and stratified according to the size of the herd. The three surveys conducted in the territory with official FMD-free status suspended estimated levels of protection against the virus between 83.7 and 95.7% for type O virus and between 80.1 and 94.2% for type A; similar results were observed, regardless of the size of the herd. No evidence of virus transmission was observed (22).

In February 2020, the WOAH Scientific Commission recommended the reestablishment of FMD-free status with vaccination with a subdivision of the area in four FMD-free areas to reduce the impact of new potential virus incursions from Venezuela (22). One booster vaccination cycle targeted at young animals in the two FMD-free zones bordering Venezuela was also suggested, along with a reinforcement of police action to mitigate the risk of illegal movement and entry of animals and livestock products (23).

## Control of FMD in Venezuela

Venezuela, Colombia, and Ecuador reported their first FMD outbreaks in 1950, 1951, and 1956, respectively and were affected by type O and type A viruses (24). Between 2001 and 2010, Venezuela exhibited an annual average of 24 FMD outbreaks, ranging from 3–63 outbreaks. As of 2006, the vaccination program was strengthened with a social plan, in which the state covered, free of charge, the vaccination of the bovine population of small stockbreeders, who had been historically excluded from the two annual vaccination cycles, which led to an increase in population immunity (25). The improvement of vaccination coverage in the population was reflected in the reduction in the number of annual outbreaks since 2009, with the last FMD outbreak reported in the state of Barinas in April 2013. Furthermore, the Venezuelan Program for the Control and Eradication of Foot-and-Mouth Disease was validated by the WOAH in 2015 (26). In the same year, a random cross-sectional sampling of herds and bovines was carried out to evaluate the prevalence of post-vaccination antibodies in two states in the southwest of the country, and the sampled population showed a satisfactory level of protection consistent with the vaccination frequency of the control program in the different categories of sampled bovines (19).

However, in 2016, Venezuela reported to COSALFA that the provision of vaccines, particularly those for the social plan, would be restricted due to the critical economic situation of the country that year (27). In 2017, the WOAH withdrew validation of the FMD program from Venezuela (28).

The deterioration of the vaccination program can be observed by comparing the average number of vaccinated bovines by year. In 2015, 15,448,097 bovines (average of the two annual vaccination cycles) were vaccinated during the vaccination cycles, corresponding to a coverage >90% of the bovine population (29), whereas in 2020, an average of only 6,358,255 bovines were reported to have been vaccinated, indicating a coverage < 50% (12). However, the absence of reports of FMD outbreaks since 2013 has a high degree of uncertainty, as the surveillance system has experienced increasing limitations that compromise its sensitivity and coverage. Moreover, serological studies have not been conducted to detect virus transmission in vaccinated bovine populations. Since 2021, efforts have been made between the public and private sectors, jointly with international cooperation, to reestablish the control program.

## Verifying the elimination of the type C FMD virus

The occurrence of FMD caused by the type C virus in South America was described by Saraiva and López (30), and more recently by Sanchez-Vazquez et al. (31), while its phylogenetic evolution in the subcontinent was described by Paton et al. (32). In 2016, 12 years after the last case of FMD caused by the type C virus, only four countries in South America (Argentina, Bolivia, Brazil, and Paraguay) kept the type C virus in their vaccines in use. Therefore, COSALFA requested PANAFTOSA-PAHO/WHO to carry out a risk assessment, at the regional level, to estimate the risk of serotype C persistence, recommending the applicable risk-management measures.

The risk assessment considered that the infection caused by the type C virus in the vaccinated bovine population might naturally occur *via* 3 routes: the environment, wild animals, and carrier cattle or endemic niches. Additionally, the risk of non-detection by surveillance activities carried out by veterinary services was evaluated (33). The probability of persistence of the type C virus in the environment was considered negligible due to the time elapsed since the last outbreak. The probability of persistence of the type C virus in wild animals was also considered negligible since no role of wild animals as reservoirs of FMD virus had been demonstrated in the South American subcontinent (33).

Because the bovine species has played the main role in maintaining and disseminating FMD in South America, evaluating the risk of release of the type C virus by carrier animals and in endemic niches of infection was of interest. Although several research projects have focused on identifying the role of carrier animals in the dissemination of FMD, it was not possible to determine whether they played a significant role in the transmission of the infection (34), and considering the time elapsed since the last outbreak caused by the type C virus, the risk was considered negligible. Moreover, evaluating the risk of persistence of endemic niches of infection is important, due to the evidence of the occurrence of sporadic FMD outbreaks caused by a genotype of the type O virus in FMD-free countries with vaccination of the Southern Cone sub-region. However, considering that presentation patterns of the type C virus are characterized by a lower prevalence of outbreaks, a more limited geographic distribution, and different temporal patterns compared to those observed with type O and type A viruses, lower transmissibility than the other virus types is suggested. Additionally, type C virus is likely to have a higher response to systematic vaccination programs, as demonstrated by the absence of recurrence of infection after withdrawal of the vaccine or the removal of the type C virus from vaccines, three to 6 years after the last outbreak. This was the case in Chile (1980), Uruguay (1994), Argentina (1999), Peru (2001), and the state of Santa Catarina in Brazil (2001). Therefore, the probability of endemic niches of infection caused by the type C virus in vaccinated populations was estimated to be negligible.

Finally, the risk of not detecting infection in vaccinated populations was assessed based on the information gathered by both passive and active surveillance systems since 2004. Clear evidence has suggested that the passive surveillance system implemented by the veterinary services in South America reaches all territories with susceptible animal populations and is specialized in attending to all cases with a suspected vesicular disease, which is supplemented with active surveillance actions, mainly regular serological surveys for the detection of virus transmission (33). Therefore, the evidence gathered by the combined passive and active surveillance systems provides high confidence of the condition of being free from infection and suggests that the likelihood of not detecting an infection caused by the type C virus in FMD-free countries with vaccination is negligible. Consequently, PANAFTOSA-PAHO/WHO suggested the suspension of the inclusion of the type C virus in the vaccines and a specific risk mitigation strategy for the stocks of the type C virus in vaccine manufacturing laboratories and diagnostic virology laboratories in the region (33).

The inclusion of the type C virus in the vaccines in use was suspended by Bolivia and Brazil in 2018 and by Paraguay in 2019 (20, 22). Argentina, which had decided to reintroduce the type C virus in the vaccines in 2004 due to the FMD outbreak in the Brazilian Amazon, still maintained this virus type in the vaccines used in the national program through the end of 2021.

## Transition to FMD-free status without vaccination from 2011–2020

The PHEFA considers the status recognized by the WOAH as the milestone to achieve disease eradication in the affected countries of South America. In 2010, 56% of herds and 81% of bovines and buffaloes were in countries and zones recognized as FMD-free with vaccination, and only 6.8% of herds and 3.4% of bovines and buffaloes were in countries and zones with FMD-free status without vaccination (4). However, 34% of herds and 15% of the bovine and buffalo population of South America had no official recognition of their FMD status. This latter group with no official recognition included Ecuador and Venezuela, which at that time were experiencing an endemic occurrence of FMD, as well as the departments of the Altiplano, Los Valles y Llanos Orientales of Bolivia, the north and northeast regions of Brazil, departments of the north and northwest of Peru, and Suriname, which had no occurrence of FMD (4).

Bolivia had recorded its last case of FMD in 2007 and had achieved the recognition of two isolated zones as FMD-free with vaccination in the Altiplano y Llanos Orientales. In 2011, the progressive official recognition of the regions of Bolivia began, and in 2012, the departments that made up the Bolivian Altiplano were recognized as FMD-free without vaccination. In 2013, a zone including the regions of Chaco and Los Valles was recognized as FMD-free with vaccination, a status which spread to the rest of the country in 2014 (11, 13). In 2018, Bolivia suspended vaccination in the department of Pando, which was recognized as FMD-free without vaccination the following year. Furthermore, vaccination was suspended in the rest of the departments with FMD-free with vaccination status in 2019, except for the department of Santa Cruz. However, until the end of 2021, no actions had been taken to achieve the official recognition of these departments as FMD-free without vaccination.

In May 2013, Peru achieved the status of being FMD-free with vaccination in a region located in the north of the country bordering Ecuador. This recognized zone was used to serve as a protection zone for the rest of the country, since at that time Ecuador was a country without recognized health status. Concomitantly, Peru completed the requirements to gain the status of FMD-free without vaccination in a zone that included departments of the northeast of the country, where vaccines were no longer used and the last FMD outbreak had occurred in 2004, thus achieving recognition as FMD-free throughout the country (35). In 2017, Peru suspended the use of vaccines in the FMD-free zone located in the north of the country and, in 2018, the whole country was recognized as FMD-free without vaccination (36).

In 2014, Brazil extended its official recognition as FMD-free with vaccination to a zone that included seven states of the northeast region and part of the state of Pará, where the last outbreak had occurred in 2003 (11). In 2018, the whole country was recognized as FMD-free when official recognition was achieved for the states of the northern region, Amazonas, Roraima, Amapá, and part of the state of Pará (36). The last outbreak in that zone had been documented in 2004 (37).

In 2017, Brazil approved the Strategic Plan 2017–2026 of its National Foot-and-Mouth Disease Prevention and Eradication Program, which established a schedule for the transition to the status of FMD-free without vaccination by means of the progressive suspension of the vaccine in the 5 blocks in which the 25 states and the federal district of the country with vaccination had been grouped (38). The goal was to recognize the whole country as FMD-free without vaccination by 2023. In 2019, the use of vaccines was suspended in the states of Paraná, Acre, Rondonia, and a group of municipalities in the states of Amazonas and Mato Grosso to which the state of Rio Grande do Sul was added at the beginning of 2020. In 2021, these zones were recognized as free without vaccination, which along with Santa Catarina (recognized as FMD-free without vaccination since 2007) comprised 20% of the bovine population of the country (23). Consequently, by the end of 2021, 1,945,161 herds (35.9% of the total) and 57,372,953 bovines and buffaloes (15.5% of the total) were in FMD-free countries or zones without vaccination in South America (12).

Suriname, a country that had never recorded an FMD outbreak, achieved all the necessary requirements for recognition as FMD-free without vaccination in 2018 (36).

In 2015, COSALFA confirmed that no new outbreaks of FMD had occurred for 3 years in the South American territories that were FMD-free, and the last stage of the PHEFA began. Thus, COSALFA approved a technical guideline with methodologies that would allow the FMD-free countries with vaccination to evaluate the risks for making a safe transition to FMD-free status without vaccination while reducing the vulnerabilities in their animal defense systems to preserve the FMD-free status (39).

## The PHEFA action plan 2021–2025

By the end of the PHEFA Action Plan 2011–2020, the percentage of South American territory officially recognized as FMD-free had increased from 67.6% in 2011 to 95.1% by the end of 2020. Moreover, the herds in FMD-free countries and zones that accounted for 63.7% of those in South America at the beginning of the Action Plan 2011–2010 increased to 98.6%, and the percentage of the bovine and buffalo population in FMD-free countries and zones increased from 84.4 to 95.8%. Nearly 1.4% of the herds and 5% of the bovine population of South America remained without sanitary recognition, including the whole territory of Venezuela in which, although no cases have been reported since 2013, the elimination of virus transmission has not been verified in the vaccinated population (39). Likewise, North America, Central America, and the Caribbean have not documented the occurrence of FMD outbreaks during the whole period as a result of an FMD prevention policy characterized by a high level of protection (40).

By the end of 2020, the risk of FMD was confined to the north of the Andean sub-region, as confirmed by phylogenetic studies conducted by Malirat et al. (8, 13), which found evidence of the circulation of specific lineages of FMD viruses in bovine populations restricted to certain sub-regions of South America, with no historic evidence of their presence in other sub-regions, thus reflecting the high degree of epidemiological independence among sub-regions (40).

Since 2011, more than 9 years have elapsed without new occurrences of FMD in FMD-free countries with vaccination (except for Colombia), compelling these countries to confirm the elimination of the virus in vaccinated populations by suspending vaccination campaigns.

In 2020, at the request of the 13 countries of COSALFA, the representatives of the six sub-regions of the Americas in COHEFA approved the third Action Plan of PHEFA covering the 2021–2025, with the overall purpose of completing the eradication of FMD in South America and strengthening the prevention and response capacity of the veterinary services of the countries in this continent. Such a goal can be achieved with actions aimed at three specific objectives: (a) eradicating the FMD virus in the territory of Venezuela and mitigating the risk in the Northern Andean sub-region, (b) making the transition to the official status of FMD-free without vaccination in the FMD-free countries still using vaccines, and (c) maintaining the status of the FMD-free territories without vaccination (40).

## Discussion

In its more than 33 years of execution, PHEFA has provided valuable insights in the efforts to eliminate FMD in South America.

First, PHEFA has had a regional governance mechanism made up of COHEFA and COSALFA, which has allowed not only coordinated actions under a master program that has guided national programs, but also permanent monitoring of progress in the elimination of the disease and a space for discussion and genuine collaboration between the public and private sectors with the support of international cooperation. As a result, the definition and implementation of regional strategies to solve problems and delays found during the execution of the program have been made possible.

Second, the adoption of oil-type vaccines accompanied by quality control of all the series in use, complying with regional and international standards, has allowed national programs to rapidly control outbreaks and eliminate infection, since the vaccination programs reached high coverage, both at the herd and population level.

Third, a pattern of sporadic outbreaks in bovine populations that reached FMD-free status with vaccination revealed the persistence of FMD virus transmission and the presence of endemic niches in vaccinated populations. These niches corresponded to sub-populations with low immunity due to lower coverage and/or bad practices in vaccination campaigns.

Fourth, the introduction of studies to measure post-vaccination immunity, not only in a generalized manner, but also to characterize it through simple indicators such as the size of the farm, the age of the animals, and the identification of geographic clusters, served to identify the failures in vaccination and introduce corrections, both in the frequency of vaccination and in its application. The review and strengthening of vaccination campaigns, including additional cycles for young animals, as was done in Colombia, Ecuador, and Paraguay, was a decisive strategy for mitigating the risk of transmission and eliminating the infection.

Fifth, the isolation of the active viruses in each outbreak of the disease has allowed a phylogenetic characterization of the different lineages of viruses present in the region, linking them to their area of occurrence, and has revealed that the transmission patterns among the sub-regions of South America (i.e., Southern Cone, Andean area, and Northwest) have been limited, demonstrating a particular segregation of risk in the region.

Finally, the movement of animals, conditional on compliance with the vaccination program, has been strengthened with the improvement of the cadaster and identification of herds supported by computer tools, which ensure centralized and effective control of the movement of each batch of animals, without requiring the individual identification of the animals, except in the case of batches intended for the export of livestock products.

The territories that have taken the step toward withdrawing the vaccine and being recognized by the WOAH as FMD-free without vaccination are contributing to the absence of virus transmission, confirming the elimination of FMD in those areas.

## Author contributions

AR led the article conception, writing, and review of this article. MS-V has shared the article conception task with AR and has contributed to the writing and review. EP and LB have contributed to the writing and review. MM and OC has contributed to the review. All authors contributed to the article and approved the submitted version.
